# Purification Methods and the Presence of RNA in Virus Particles and Extracellular Vesicles

**DOI:** 10.3390/v12090917

**Published:** 2020-08-21

**Authors:** Yijun Zhou, Ryan P. McNamara, Dirk P. Dittmer

**Affiliations:** 1 Lineberger Comprehensive Cancer Center, The University of North Carolina at Chapel Hill School of Medicine, Chapel Hill, NC 27599, USA; yijun.zhou@med.unc.edu (Y.Z.); ryanpm@email.unc.edu (R.P.M.); 2 Department of Microbiology and Immunology, The University of North Carolina at Chapel Hill School of Medicine, Chapel Hill, NC 27599, USA

**Keywords:** virion RNA, extracellular vesicles, exosomes, herpesviruses

## Abstract

The fields of extracellular vesicles (EV) and virus infections are marred in a debate on whether a particular mRNA or non-coding RNA (i.e., miRNA) is packaged into a virus particle or copurifying EV and similarly, whether a particular mRNA or non-coding RNA is contained in meaningful numbers within an EV. Key in settling this debate, is whether the purification methods are adequate to separate virus particles, EV and contaminant soluble RNA and RNA:protein complexes. Differential centrifugation/ultracentrifugation and precipitating agents like polyethylene glycol are widely utilized for both EV and virus purifications. EV are known to co-sediment with virions and other particulates, such as defective interfering particles and protein aggregates. Here, we discuss how encased RNAs from a heterogeneous mixture of particles can be distinguished by different purification methods. This is particularly important for subsequent interpretation of whether the RNA associated phenotype is contributed solely by virus or EV particles or a mixture of both. We also discuss the discrepancy of miRNA abundance in EV from different input material.

## 1. Introduction

Virus particles have similar biophysical attributes to naturally occurring extracellular vesicles (EV) [1]. Virus particles, or virions, have been studied for over 100 years and continue to be the subject of intense interest in the fields of basic sciences and vaccine manufacturing [2]. Hence, we use virions as a point of comparison and frame of reference for this review. We will discuss questions central to the packaging and delivery of RNAs by EV from foundational knowledge learned through virus infection. Specifically, we will consider the following questions:

First, how do RNAs co-purify with viruses and with EV by different purification methods?

Second, what have we learned from different purification methods? Do we need standards and, if so, what criteria should be used to evaluate claims of RNA-EV and RNA-virion associations?

Third, do DNA viruses package miRNAs or mRNAs into virus particles [3]? All viruses package their respective genomes into an inner protein shell, the capsid, in a targeted, coordinated, and well-controlled process. For DNA viruses, mature virion preparations have been repeatedly found associated with RNA [4,5,6,7,8,9,10,11,12,13,14,15]. For RNA viruses as well, RNAs other than full-length genomic RNA have been reported in mature virion preparations. Is it possible that EV may have contaminated the virion preparations in these studies? Has there been enough experimental evidence to rule out EV contamination?

Forth, how many RNAs on average does each EV contain? How do experimental models affect the heterogeneity of results? How can EV induce a physiologically significant response while current evidence shows the encased copies of RNAs are far from enough? Upon release, EV are rapidly diluted in solution. Elementary physics posits that Fick’s laws govern EV concentration, diminishing inversely to the cube of distance (1/d^3^). Fick’s laws apply to all experiments conducted in culture dishes and represents a lower limit regarding experiments in animals, where inter-tissue diffusion, intra-tissue diffusion, clearance, and blood flow rapidly dilute bolus injections of EV. Viruses overcome physiological dilution by utilizing highly specific receptors and co-receptors, like cluster of differentiation 4 (CD4) [16] and C-C chemokine receptor type 5 (CCR5) [17] on T cells in the case of human immunodeficiency viruses (HIV), and by being able to replicate in infected cells, thereby amplifying the signal locally. Should we postulate similar, target-cell specific mediated entry for EV, or is the concept of a single infection event, EV bolus the wrong experimental paradigm? 

**Figure 1:**
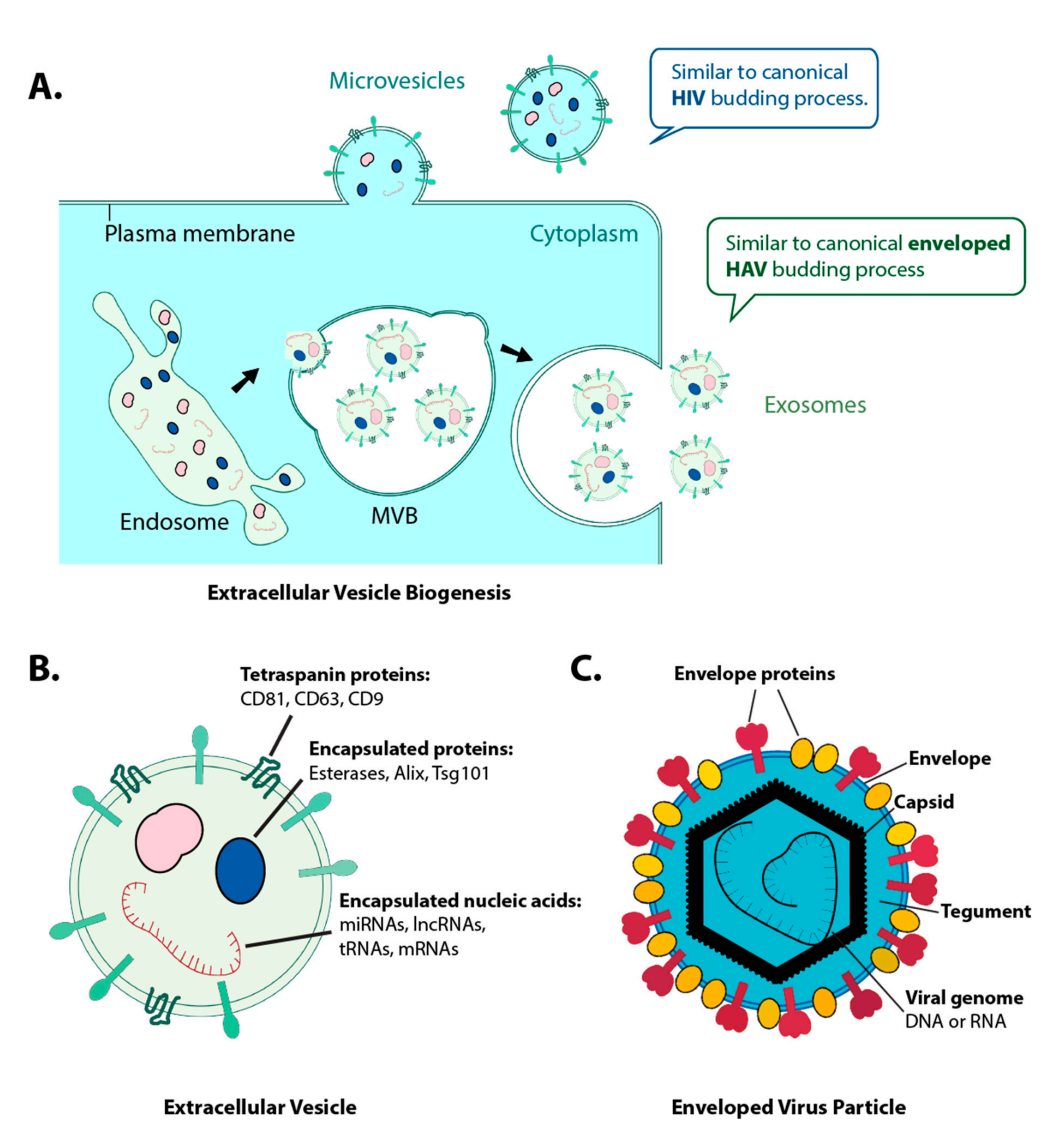
Extracellular vesicles (EV) and virus particles share similar vesicular budding process and composition, including proteins, nucleic acids, and lipids. (**A**) Microvesicles bud off at the plasma membrane, similar to the canonical human immunodeficiency viruses (HIV) budding process. Exosomes originate from inward budding of the late endosome into multivesicular body (MVB) and later release at the plasma membrane, similar to the canonical enveloped hepatitis A viruses (HAV) budding process. (**B**) Extracellular vesicles may carry makers like tetraspanins, esterases, Alix, and Tsg101 [1]. The encased nucleic acids are protected from nucleases. (**C**). A virus particle consists of an envelope, capsid, tegument, and viral genome.

We will start our discussion by defining some key terms. EV are divided into three major classes: (i) apoptotic bodies, (ii) shedding microvesicles, and (iii) exosomes [1,18]. These EV have historically been classified based on their origin, content, and size. (Figure 1) Apoptotic bodies result from cells undergoing apoptosis and can thus contain any part of those cells, including chromatin and mitochondria. They vary considerably in size, with some reaching several micrometers (µm) in diameter and are generated any time that cells are grown in culture or as part of an internal organ. Their concentration in cell supernatant can vary by orders of magnitude depending on the health of the cell at the time of harvest. Microvesicles bud off at the plasma membrane and become enriched for outer membrane-associated proteins, soluble proteins, and metabolites present in the cytosol. Microvesicles range in sizes from 80–500 nanometers (nm) and are less dense than apoptotic bodies. Lastly, exosomes originate from inward budding of the late endosome into the multivesicular body (MVB) [1]. Exosomes range from ~40–150 nm in diameter and are enriched for traffic proteins of the MVB, like the endosomal sorting complex required for transport (ESCRT) proteins, Alix, and tetraspanins such as CD63, CD81, and CD9 [1,19,20]. Exosomes are found in high concentrations (>10^8^/mL in cell culture systems and >10^9^/mL of body fluids such as plasma and urine) [21,22] and exhibit wide heterogeneity among biological fluids [18] Their particular intracellular origin make exosomes distinct from the other classes of EV; however, EV with the same size and biophysical characteristics as exosomes can also bud from the plasma membrane (reviewed in [23]). Proteins, DNA, mRNA, miRNA, and other non-coding RNAs were found enclosed in these small, membrane-enclosed exosomes and microvesicles [1] (Figure 1B). This makes EV conceptionally and biochemically similar to viruses [1,18] (Figure 1C). The formation and egress of microvesicles and exosomes share similarities to virus biogenesis, such as HIV [24] and enveloped hepatitis A viruses (HAV) [25] (Figure 1), respectively. In fact, viruses have been thought of as emerging from exosomes or vice versa [26]. Any EV can play an analogous role to a virus particle in the functional transfer materials from one cell to the next, regardless of the class. 

## 2. How do RNAs Co-Purify with Viruses and EV by Different Purification Methods?

Viruses and EV are purified by similar techniques (Table 1) [18,27]. Historically, differential centrifugation and ultracentrifugation have been the most widely used methods for concentrating viruses and EV [28]. RNAs co-purify with viruses and EV in the form of (a) non-encased extracellular RNAs and (b) co-contamination of RNA encased in EV and virions. For cleaner purification, RNase treatment are always recommended to remove non-EV encapsulated extracellular RNAs. Co-purified RNAs within a heterogeneous mixture can obscure definitive and functional research on EV as well as viruses. Separating virions from EV, however, is much more challenging. Thus, we will discuss how such contamination—if any—can be removed by different purification methods. 

Ultracentrifugation—Centrifugation separates particles based on density and size. The standard equation for sedimentation velocity is:

$$v=\frac{dr}{dt}=\frac{d_{p}^{2}\left(\rho_{p}-\rho_{m}\right)w^{2}r}{18\eta }$$

Here, $d_{p}$ indicates particle diameter; $\rho_{p}$: particle density; $\rho_{m}$: medium density; $w^{2}r$: centrifugal force; η: viscosity of the medium [29]. When the differences between particle sizes ($d_{p}$) and densities ($\rho_{p}$) are big enough, i.e., between soluble protein and cells, they are not likely to contaminate each other. When the differences are small, i.e., between viruses, EV and protein aggregates [30], a gradient medium (i.e., sucrose, iodixanol, sorbitol, cesium chloride, etc.) is needed to increase the separation efficiency [29]. Often the viruses’ densities and buoyancies so closely overlap with exosomes’, that even separation via density gradients is impractical [18,31,32,33] and leads to co-isolation of their encased RNAs.

Polyethylene glycol (PEG) Precipitation **-** PEG has long been used to “precipitate” and purify viruses [34,35]. It is the main reagent in several commercial kits for exosome purification [18,35]. The method consists of a precipitation step followed by low-speed centrifugation. PEG precipitation offers little separation efficacies, cannot separate viruses from EV [36], and often co-precipitates other macromolecule contaminants like RNA, DNA, and protein aggregates [30,35,37,38]. Exosomes isolated by commercial kits are likely to be contaminated by viruses, proteins, non-EV associated nucleic acids, and other extracellular debris [36]. This includes any molecules stuck to the outside of the EV rather than being carried inside. Many of these contaminations may carry RNAs. 

Filtration**-**filtration is a size-based separation technique. Based on pore sizes of the membranes, filtration is divided into microfiltration (0.1–1 µm) and ultrafiltration (0.01–0.1 µm). Ultrafiltration membranes are referred to according to their molecular weight cutoff (MWCO). The technique can be performed either by normal flow filtration (NFF) or by tangential flow filtration (TFF), also called cross-flow filtration [39]. It separates particles of different sizes, and—equally important—monodispersed particles from aggregated particle assemblies. For instance, a typical herpesvirus virion is approximately 180 nm in size, but virions of many viruses as well as EV, tend to aggregate under conditions of high particle density. These aggregates can reach almost the size of bacteria, and thus can be filtered with 0.22 µm microfilters to separate from single EV (50–150 nm). In sum, separation of virus and EV and their encased RNA by filtration involves empirical process optimization, depending on the characteristics of viruses and EV [18,40]. 

Chromatography- Size-exclusion chromatography (SEC), ion-exchange chromatography (IEC), and affinity chromatography (AC) are commonly used chromatography methods for virus purification [41,42,43]. In SEC, the smaller, soluble proteins and nucleic acids are retained by the resin, while the larger particles such as viruses or EV migrate much faster and can be recovered in the flow-through. In IEC, separation is based on charge. Anion-exchange resins retain negatively charged particles and cation-exchange resins retain positively charged particles. In general, SEC removes protein and DNA, while IEC mainly removes DNA (by anion-exchange) [44]. SEC and IEC are often used in tandem at the sample polishing step, between the preceding clarification and concentration (i.e., by microfiltration, centrifugation) and final concentration (i.e., TFF, centrifugation) steps [43,44]. Both viruses and EV can be purified by SEC and IEC [18,40] but virus-EV cross-contamination is difficult to avoid [30]. Thus, SEC and IEC will most likely co-isolate the encased RNAs contaminants. 

Affinity chromatography (AC) offers a higher separation efficiency. AC relies on specific ligands, either low molecular weight or antibodies. For instance, heparin chromatography separated HIV-1 virus like particles (VLPs) from EV, after a Capto Core 700 polishing step [45]. In bead-based affinity purification, magnetic beads are coated with antibodies specifically targeting exosomal markers (tetraspanins, CD9, CD63, and CD81). Of note, Pegtel and Gould [23] have proposed that the canonical exosome marker CD81 is more enriched on the inner leaflet of the plasma membrane than in the endosomal trafficking network, meaning that affinity purification using anti-CD81 beads can purify what has previously been considered both exosomes and microvesicles. Bead-based purification efficiency depends on antibody affinity, availability, and ligand density on viruses or EV surface [30]. This technique rapidly and successfully enriched and separated marker-positive EV from viruses, and other EV which did not carry the specific marker and allowed the identification of exosome enclosed RNAs and proteins [32,33,36,46,47]. 

## 3. What Have We Learned from Different Purification Methods?

Ultracentrifugation is widely used to purify EV [28]. This method originates from the study of viruses and intracellular compartments such as the Golgi apparatus or the endoplasmatic reticulum. An iodixanol prepared gradient was successfully used to isolate HIV [48], encephalomyocarditis virus (EMCV) [25], and herpes simplex viruses 1 (HSV-1) [49] and later to separate virion particles from EV and different density EV fractions from each other. Separations of virion and EV were confirmed by testing for the presence of virus capsid and envelope proteins [48,49], infectivity [25,49], and the presence of EV markers CD63, CD9 and acetylcholinesterase for EV fractions. EV from different iodixanol density gradient fractions showed diverse protein and RNA contents [50,51] (Figure 1B), reflecting the heterogeneity among subpopulations of EV [30] or contamination of EV with cellular compartments after cell death (apoptosis, necrosis, nepotosis). High sedimental force (30,000–100,000 g) is required due to the low density of EV. Such high sedimental forces can only be provided by ultracentrifuges, which is limited by volume inputs. Repeated ultracentrifugation increases purity but decreases the yield and quality of the purified EV [38,40]. 

PEG-based methods, such as ExoQuick (System Biosciences, LLC) effectively precipitate EV out of solution. They also, invariably, precipitate contaminant soluble proteins and PEG concentrates almost all viruses. For high throughput diagnostics, this is completely acceptable as it maximizes analytic sensitivity at the cost of specificity; however, EV isolated by precipitation alone is not suitable for functional studies. Of note, PEG can change the osmolarity inside a vesicle by creating external osmotic pressure but PEG itself does not cross the lipid membrane [52].

Proteinase digestion [53] can remove proteins associated with EV or with virion particles. This changes their buoyant density. Thus, proteinase digestion can be added as an extra step before centrifugation to separated subfractions of EV and EV from viruses. It does, however, also shave off the extracellular domains of any membrane-associated proteins, which in the case of viruses are responsible for receptor-mediated endocytosis [54].

Nucleic acid digestion removes molecules that are attached externally to the EV, such as circulating RNAs and cell-free DNA (cfDNA) fragments. RNase treatment represents a classic and essential step in separating externally associated RNAs from encased and protected RNAs. Of note, certain miRNAs that are associated with Ago proteins, but not part of EV are protected from RNase treatment [55]. While externally carried RNAs, on EV or virions, may have diagnostic value, they are subject to extensive and random RNase degradation, much like circulating cell-free DNA (cfDNA). It appears unlikely that externally carried RNA would enter the cytosol or the same compartments of the target cell as other EV cargo. Hence, it is at present unclear how these free-floating molecules would have a defined biological function. 

In our hands, filtration by a larger-than-particle diameter is essential in removing aggregate EV, fused vesicles, and virions. For instance, filtration by 0.22 µm microfilter was essential in separating Kaposi’s sarcoma herpesvirus (KSHV) virion from KSHV–EV [36]. The availability of an internal standard was the key to this experiment. For this particular virus and for herpesviruses, each particle carries exactly one DNA molecule. DNA molecules are incorporated into the virus capsid already inside the nucleus, before assembly in the cytosol. Unfortunately, a similar maturation standard does not exist for EV. EV take-up RNAs from the cytosol or at the plasma membrane. In the case of miRNAs, this requires processing by dicer after the nuclear egress of pre-miRNA into the cytoplasm [56]. Hence, separating the viral DNA signal from the viral miRNA signal served as a highly sensitive measure of EV purity. 

Commercially available SEC columns, such as qEV (Izon Science, LTD), outperform other precipitation-based commercial kits according to particle/protein ratios [38]. Recently, a Capto Core resin was introduced to combine SEC and IEC purification on the same resin [57,58,59]. For instance, Capto Core 700 has a ligand-activated core enclosed within an inactive porous shell that has an MWCO of 700 kDa. The activated core binds to soluble protein or DNA and the porous shell excludes viruses or EV, resulting in a high recovery in the flow-through with limited contaminants.

A very recent example [60] used asymmetric flow field-flow filtration (AF4) and successfully purified subpopulations of EV—large (90–120 nm), small (60–80 nm) and non-membranous particles(~35 nm)—based on size and molecular weight.

Chemicals and proteins may be used to inhibit EV secretion (or virion maturation) and thereby reduce contamination of virion preparations or EV preparations. For example, the neutral sphingomyelinase (nSMase) inhibitor GW4869 [61,62] and the TAT-5 phospholipid flippase and its regulator proteins [62,63] can inhibit EV secretion in some cell lines. Ionophores like ionomycin [64] and monensin [65] can induce EV secretion and thereby increase the yield. 

Affinity-based tools and specialty column resins provide this capability and often out-perform traditional methods. Follow up experiments such as Western blot for virus or EV protein markers, virus plaque assay for infectivity or ELISA for EV marker acetylcholinesterase activity have been used to confirm separation of the virus from EV. The field standard [28], written by the International Society of Extracellular Vesicles (ISEV), can be used as a reference. 

In sum, separating virions from EV is highly dependent on the intended use (structural studies, functional studies, diagnostic), the density, and the size of the specific EV populations and virus species that are being investigated. More than one technique is typically needed to ensure the separation of these two biologically different populations [38,40,60]. 

## 4. Do DNA Viruses Package RNAs in Virus Particles?

A virus particle acts as an extracellular vehicle to transfer the viral genome, virus-encoded proteins and enzymes, and some host factors. RNA viruses package RNA, in the form of the viral genome, but also for other purposes. For instance, HIV packages transfer RNA (tRNA) molecules in each virion to initiate reverse transcription [24,66,67]. The HIV retroviral Gag protein can assemble into virus-like particles, with or without the viral genome [68] with a roughly normal amount and nearly a random sampling of the RNA in the cytoplasm. Gag binds to the “packaging signal” (ψ) in the viral genome but also has high affinity to other RNAs [69]. tRNA is the best characterized virion-encased RNA for HIV [70]. Other RNAs [71,72,73] were also found to be encased in HIV virion. Interestingly, the possibility of EV contamination in the HIV virion preparations has not been ruled out in these early reports. Here, we used DNA viruses as an example to discuss why it is important to verify if EV is present in the virion preparations. Both mRNAs and non-coding RNAs were found in purified herpesvirus and adenovirus virions [4,5,6,7,8,9,10,11,12,13,14,15] (Table 2), even though herpesvirus and adenovirus are double-stranded DNA viruses and RNA intermediates are not involved in viral replication. Despite many studies, the mechanisms for incorporating mRNA and non-coding RNA into virions remains incompletely understood and is subject to intense debate. The biological roles that the EV-mediated transfer of RNAs plays in viral pathogenesis are just beginning to emerge, for example in human herpesviruses [36,46,74,75,76]. Insights derived from the virus-mediated transfer of intact RNAs could, therefore, provide an example for EV-mediated transfer of functional RNA.

Separating EV from virus particles, particularly exosomes and microvesicles, has proven to be a considerable hurdle in the field of host–pathogen interactions. Chugh et al. [36] and Bess et al. [77] showed that virions and EV co-sedimented in various isolation techniques due to their similar size, density, and sedimental velocity [31]. Other studies [25,27,31,32,36,48,77] also showed that neither differential centrifugation nor commercial exosome precipitation reagents separate virions from EV. In some instances, this was possible, and several groups [25,48,49] have developed finely tuned protocols for EV vs. virion separation using density gradient centrifugation with iodixanol. Iodixanol gradient centrifugation was not used in the studies listed in Table 2. These twelve studies generally included a low-speed (1000–4000 rpm) centrifugation step to clear cell debris, followed by a high-speed (>20,000 rpm) cushioned/gradient centrifugation step, and a final high-speed (>20,000 rpm) centrifugation to concentrate the material. Two studies [9,12] included 0.4 µm filtration but did not discuss if it was adequate to separate virions from EV. Eleven studies [4,5,6,7,8,9,10,12,13,14,15] treated the virion preparations with RNase or other nucleases. Since the EV encased RNAs are also protected from digestion, it remains a lingering concern that the RNA species, which were ascribed to virions, may be carried by co-contaminating EV rather than the virions themselves. 

Breshnahan, et al. [14] was aware of possible EV contamination. Their virion preparations were centrifuged through a tandem, three step centrifugation—sorbital cushion, glycerol–tartrate gradient and CsCl gradient—to ensure a single fraction of narrowly defined density was harvested.

Greijer et al. [7] treated the purified virions with detergent, which disrupts EV, in the presence of RNase A and DNase I, and still detected mRNA in the HCMV capsids. However, much less RNA can be isolated from capsids compared to the same number of virions. This represents a special case of RNAs residing in the viral protein shell, which is inside the lipid-encapsulated virion. Even in this special case, the majority (>90%) of co-purified RNAs were inside detergent-sensitive vesicles. They could be between the capsid and the outer virion envelope or they could have been in co-purified EV.

Since it is near impossible to separate EV from virions by biochemical methods, the absence of EV is typically demonstrated by the absence of EV protein markers. For instance, Cliffe et al. [8] checked the purity of their virion preparations by transmission electron microscopy, but no image was included in the manuscript. Lin et al. [9] performed a Western blot and did not detect the exosome markers CD63 or CD81 in the purified virions, concluding that miRNAs were present in virions. However, whether the Western blot had the required level of sensitivity is unknown. In contrast to Lin et al., Chugh et al. [36] showed that for the same virus, the majority of miRNA are carried by EV rather than virions. Herpesvirus can switch between latent and lytic phases [78]. The RNA profiles are very different [79,80,81]. It is not clear whether the phase of the virus played a role in the discrepancy between Lin et al. [9] and Chugh et al. [36]. The majority of the studies in Table 2 [4,5,6,7,10,11,12,13,15] did not investigate possible EV contaminations. The concept of EV transferring functional nucleic acids has only gained traction recently [82], so it is not surprising that studies before 2010 did not consider this possibility.

The problem becomes more difficult when considering that as virus-infected cells not only release virions with virus-derived RNAs, they also release EV filled with virus-encoded RNAs at the same time, as well as various species of defective interfering particles. Hence, we would expect EV to contain viral RNA under most circumstances. EV emanating from cells infected with HIV, hepatitis C virus (HCV), and various human herpesvirus viruses (HHV) can have virus-encoded RNAs present within them [83,84,85]. In the case of KSHV, viral miRNAs are present predominantly within exosomes, rather than mature virions [36]. Additionally, picornavirus like the EMCV and HAV can traffic the entire virion into EV [25]. 

## 5. How Many miRNAs are in an Exosome?

Another open question in the field is how many copy numbers of nucleic acids are present in a single EV. On the one hand, functional studies clearly show distinct phenotypes that are mediated by miRNA transfer through EV [36,75,86,87,88]. On the other hand, biochemical studies found very low levels of miRNAs per EV. This makes miRNA-mediated gene regulation by a one-time transfer of a limited number of EV unrealistic [21,89]. How can these opposing observations be reconciled?

The miRNAs, mRNAs, and other RNAs enclosed within EV are transferred to other recipient cells to elicit functional impacts [86,87,88]. The key to interpreting these studies is being able to distinguish between transferred RNAs and RNAs that are synthesized in the recipient cell. As all human cells can, in principle, transcribe all human RNAs and miRNAs, this is a difficult problem. Tracking viral RNAs represents an exceptionally sensitive model system to study the transduction of RNAs by EV, because viral miRNAs are only present in the originating cells but can be transferred by EV into uninfected target cells [75]. For instance, high levels of EBV BHRF1 and BART cluster viral miRNAs were transferred to monocyte-derived dendritic cells to repress target genes [75]. In KSHV infected cells, viral miRNAs can modulate the tumor microenvironment by shifting the metabolic patterns toward aerobic glycolysis [90] and can induce long-term endothelial cell reprogramming [46]. HCV-infected hepatocytes secrete miRNA-containing EV to mediate the activation of hepatic stellate cells (HSC) that cause liver fibrosis [84,85]. 

It is difficult to establish the physiological significance of EV-transduced miRNAs, without knowing how many miRNAs are transduced in each experiment. On the one hand, stoichiometric analysis of exosomes isolated from healthy or cancer patient human biofluids found a very low number of copies (0.00825 ± 0.02) of any single miRNA per EV [21,89]. On the other hand, an enriched level of certain miRNAs was found in EV from tumor-bearing mice [91]. In an attempt that calculated the copy of miRNA to be much lower than 1 copy per EV [21], exosomes were purified by a 120,000× *g* spin for 70 min. Ultracentrifugation or filtration alone may not have been enough to produce clean exosome preparations in these studies. The exosome particle numbers could be overestimated since protein aggregates or cell debris may be present and counted by nanoparticle tracking analysis [92]. Another study that reported low copies of miRNA per EV used a tandem filtration with 2, 0.8, 0.22, and 0.02 µm filters [89]. Since Wei et al. [89] isolated RNA from filter membranes, it is possible that not all RNAs may have been recovered. Differences across experiments and cell lines were also observed when trying to establish a CRISPR-Cas9 based EV–RNA transfer reporter system. HEK293T, HMEC-1, and hTERT-MSC cell lines had very diverse miRNA activity in EV [93]. EV from prostate cancer patients or other cancers may naturally contain low levels of miRNAs [21,89], but mounting evidence suggests that EV-encased nucleic acid profiles are altered in patients with various tumors [94].

**Figure 2:**
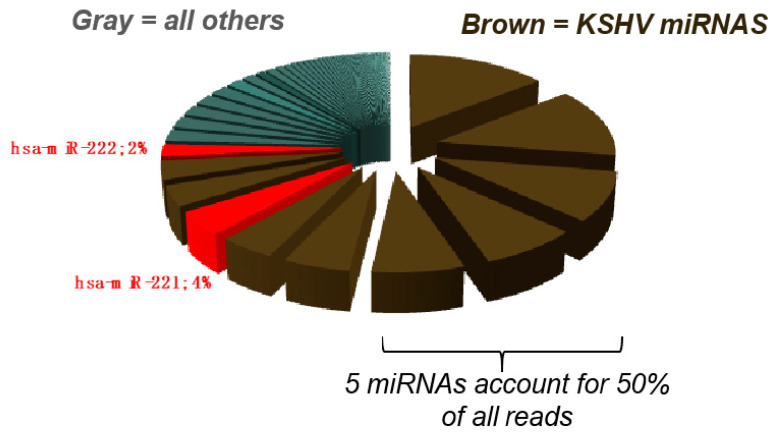
70% of all miRNAs in infected human vein endothelial cells (HUVECs) come from KSHV.

For viral RNAs, we estimate ≥ 1 miRNA per EV, i.e., comparable to liposome-mediated transfection of small interfering RNAs (siRNAs). This enrichment is due to the fact that viruses reprogram the infected cells to preferentially expressed viral RNAs that are then packaged into EV. For instance, herpesviruses are unique in encoding and expressing multiple miRNAs [95,96,97,98]. KSHV encodes a total of 12 miRNAs which constitute more than 70% of all miRNAs in infected cells and act in synergy on cellular targets [31,36,74,75,90,94,98] (Figure 2). Hence, it is likely that EV from virus-infected cells carry physiologically relevant levels of viral miRNAs. 

Unlike siRNA transfection in culture, which is a one-time event, EV mediated delivery of miRNA in vivo is a continuous and dynamic process over an extended period in a biological environment rich in EV. A single cell may be exposed to one million EV or more fusion events per cell division cycle. The precise rate of EV uptake per cell is unknown, but the EV concentration in conditioned media and bodily fluid is around 10^8^–10^9^ particles/mL [21,40] and 10^11^–10^12^ particles/mL [47], respectively. EV are also constantly replenished [47] by cells nearby or from a long distance away through the circulation system, thus exchanging biomolecules with the extracellular environment. The common cell concentration in culture dishes is around 10^5^–10^6^ cells/mL. There are about 10^9^ red blood cells, 10^8^ platelets, and 10^6^ white blood cells per mL in human blood. Roughly estimating, EV concentration is 100–1000 times higher than the nucleated cell concentration in culture and in the blood. In the meantime, EV is taken up by cells every hour [47], so over a long time such as in the case of cancer metastasis [92] or latent virus infection [31]—the cellular uptake of EV encased RNA is not negligible, especially when considering some EV can be preferentially taken up by certain types of cells [99]. 

Whereas the biological function of EV is still far from understood and requires further study, the diagnostic utility of EV is well established. Exosome enclosed miRNAs serve as potential biomarkers since miRNAs within the exosomes are protected from RNase digestion and are more stable in primary fluids than free circulating RNAs. They are especially useful for diagnosing viruses, which establish long-term low-level infections and chronic disease states that are dependent on the viral miRNAs [36,46,100]. Another chronic disease state is cancer. Here, miRNA-based diagnostics hold great promise [94,101,102], as the miRNA profile often changes at different stages of diseases and in response to drug treatment [103,104,105]. Changes in the contents of producing cells will lead to changes in the EV compositions. This is important because chronic diseases can be asymptomatic and lack biomarkers of progression. The abundance of EV in body fluids make them ideal for non-invasive diagnosis and prognosis.

## 6. Concluding Remarks

The observation that nucleic acids and proteins can be encased and transferred by EV has prompted the discovery of new interplays between host cells, host organs, and viruses. Still, better and more carefully validated purification methods are necessary to prepare cleaner virion and EV preparations before many of the proposed biological functions that have been associated with EV can be accepted. Affinity reagents, in particular, reveal surprising heterogeneity amongst EV. Describing and limiting the increasing complexity of EV may seem burdensome, but it is essential for establishing biological relevance.

## Figures and Tables

**Table 1:** Methods commonly used to purify virus and exosome are summarized based on their separation/ concentration efficiencies and scale range.

Methods	Separation	Concentration	Scale Range
Ultracentrifugation	+++	+++	5 to 250 mL
Normal flow filtration	+	++	0.5 to 1000 mL
Tangential flow filtration	+	++	100 to 5000 mL
Precipitation	-	+++	0.2 mL to >3 L
Size exclusion chromatography	+	-	0.5 mL to >3 L
Ion exchange chromatography	++	+	0.5 mL to >3 L
Affinity purification	++++	++	0.5 mL to >3 L

**Table 2:** Studies showing that RNA co-isolated with virion preparations were classified based on the purification methods used. * When indicated, the corresponding experiments were performed intending to rule out EV contamination. HSV-1: herpes simplex viruses 1, HCMV: human cytomegalovirus, MHV-68: murine gammaherpesvirus 68, KSHV: Kaposi’s sarcoma herpesvirus, HBV: hepatitis B virus, EBV: Epstein-Barr virus, WB: Western blot.

Purification Method	Gradient or Size Limit	Reference	Virion/EV Separation *	Virus	Detected RNA
Dextran gradient centrifugation	1.04–1.09 g/cm^3^	[4,5]	-	HSV-1	mRNA
Sucrose gradient centrifugation	35%, 30%–60%	[6]	-	HCMV	vRNA
	20%–40%	[7]	Detergent treatment, Infectivity	HCMV	mRNA
	20%, 10%–55%	[8]	-	MHV-68	vtRNA
	30%–60%	[9]	Banding, WB	KSHV	miRNA, usRNA
Histodenz gradient centrifugation	20%–35%	[10]	-	KSHV	mRNA
CsCl gradient centrifugation	n.a.	[12]	-	Adenovirus	mRNA
		[13]	-	Adenovirus	mRNA
Sorbitol cushion centrifugation	20%	[11]	-	HBV	miRNA
Sorbitol cushion, Glycerol-tartrate gradient, CsCl gradient centrifugation	n.a.	[14]	Banding	HCMV	mRNA
Filtration	0.8 µm	[15]	-	EBV	mRNA, non-coding RNA
